# Case Report: CD8+ T-Lymphocyte Deficit: A Prerequisite for *Pasteurella* spp. Infection?

**DOI:** 10.3389/fmed.2021.668976

**Published:** 2021-04-27

**Authors:** Eric Denes, Fabrice Fiorenza, Mateo Armendariz, Christian Martin

**Affiliations:** ^1^Infectious Diseases Department, ELSAN Polyclinique de Limoges, Limoges, France; ^2^Orthopaedic Surgery Department, Limoges Teaching Hospital, Limoges, France; ^3^Bacteriology Laboratory, Limoges Teaching Hospital, Limoges, France

**Keywords:** *Pasteurela multocida*, CD8 lymphocytes +, deficit, infection - immunology, dog bite, cat bite

## Abstract

**Background:** Immunity against *Pasteurella* spp. is not well-known for humans.

**Methods:** We've tested T CD8+ lymphocytes in a patient with a chronic prosthetic joint infection due to *Pasteurella* spp. to search for a deficit which could have favored her infection. As this deficit was found, we've searched for such a deficit in other patients with *Pasteurella* spp. Infections, either acute or subacute.

**Results:** Eight patients were tested and all had a persistent T CD8+ lymphocytes deficit. This is striking as these cells are involved in the response to this type of infection in animal models.

**Conclusion:** The authors suggest that a deficit in CD8+ T lymphocytes can be one of the causes for the onset of infections with *P. multocida*.

## Introduction

*Pasteurella* spp. is widely found in animal's mouths and is transmitted to humans by bites (1). One of the main diseases caused by this bacterium is hand tendon sheath infections. Clinical symptoms are usually acute in the first 24 h (2). Orthopedic prosthesis infections due to this bacterium are scarce and <35 cases have been reported in the literature (3).

## Case Report

We took in charge a 77-year-old patient with a total hip prosthetic infection due to *P. multocida*. Interestingly, a cat bite occurred 1 year prior to diagnosis and the patient had been complaining of pain in her left hip after the bite. Infection was treated using a two-stage surgery associated with a 6 weeks course of antibiotics (ceftriaxone and ciprofloxacin). With this management, after a follow-up of 2 years, we've conclude to a cure of this infection. This unusual chronic presentation led us to look for a possible unknown immune deficit. As we previously described a case of chronic wound skin infection due to *Rhodococcus equi* facilitated by a CD8+ T lymphocytes deficit (4), we looked for the same kind of deficit for this patient. Analyses revealed a CD8+ T lymphocytes deficit for both the percentage (15.5% - normal range: 31–40%) and the absolute value (205/mm^3^ – normal range: 500–900/mm^3^). As a CD8+ T lymphocyte deficit can be the consequence of infections, a control was performed 4 months later, when the clinical evolution was favorable and antibiotics had been stopped for 2.5 months. The deficit was still noticeable (absolute value: 220/mm^3^ and percentage: 17%). We then wondered if this deficit could play a role in the occurrence of this usually acute infection. Therefore, in a preliminary study, we performed T CD8+ lymphocytes analysis for other patients with acute *Pasteurella* spp. infection (all patients gave their consent for this analysis). First, we randomly picked up two patients who previously presented an acute hand tendon sheath infection among patients who presented this type of infection during the past 4 years. In addition, during the same period of time, several patients were hospitalized and presented acute and sub-acute infections due to animal bites. The characteristics of these consecutive patients are summarized in Table 1. Two of them (patients 7 & 8) with negative bacteriology samples, were treated with antibiotics (amoxicillin—clavulanate acid) prior to surgery, but clinical presentation was compatible with *Pasteurella* spp. infection with an acute onset in the first few hours after the bite. All patients with *Pasteurella* spp. infections had a CD8+ T lymphocytes deficit in percentage and 6 out of 8 had a deficit in absolute value. None of them presented a deficit in total lymphocytes count. The other biological parameters such as white blood cells, liver and renal function, inflammatory parameters were normal at the time of evaluation. None of them had a history of unusual infections.

**Table 1:** Summary of patient's characteristics.

**Patient**	**Age (year)**	**Sex**	**Infection**	**Bacterium**	**Type of infection**	**Lymphocytes (/mm^**3**^) Normal ranges: 800–4,000**	**CD8+ absolute value (/mm^**3**^) Normal ranges: 500–900**	**CD8+ percentage Normal ranges: 31–40**	**CD4+/CD8+ ratio Normal ranges: 1–2**	**Delay between infection and dosage (months)**
1	77	F	Total hip arthroplasty infection	*P. multocida*	Chronic	1,292	220	17	2.9	4
2	64	F	Hand tendon sheath infection	*P. multocida*	Acute	3,009	620	20	2.8	18
3	47	F	Hand tendon sheath infection	*P. multocida*	Acute	2,420	395	16.3	3.7	38
4	27	F	Osteitis	*P. canis*	Sub-acute	1,935	320	16.5	3.0	2
5	38	F	Hand tendon sheath infection	*P. canis*	Acute	1,511	413	27	2.5	3
6	67	M	Hand tendon sheath infection	*P. multocida*	Acute	2,407	503	20.9	1.9	1
7	46	F	Hand tendon sheath infection	None (antibiotics for 3 days before surgery)	Acute	2,693	482	17.9	3.2	1
8	59	F	Hand tendon sheath infection	None (Antibiotics for 2 days before surgery)	Acute	1,976	481	24.4	2.4	1

## Discussion

Although pathogenicity of *P. multocida* relies on several virulence factors among which its capsule, adhesins or toxins (5), immunity against *Pasteurella* spp. is not well-known in humans (2, 6). Antibody secretion was demonstrated against capsular and somatic antigens, however its real impact is not well-established (7). In veterinary medicine an increase of CD8+ T lymphocytes after a challenge with *P. multocida* has been demonstrated (8–10). These studies were performed with lung infection models in three different types of animals: swine (8), calves (9) and pigs (10). These studies indicate that at least concerning animals, the inflammatory response is mediated via lymphocytes activation and particularly CD8 + T cells.

*P. multocida* is the most commonly isolated pathogen after dogs or cats' bites; however it is unusual to develop an infection. Nevertheless, it is difficult to assess whether people are not infected because of their immune system (efficient CD8+ T lymphocytes for example) or because of local treatment, antibiotics or the absence of such bacteria in animals' mouth.

However, it is striking to note that 8 patients with an unusual infection (prosthetic infection), randomly chosen or consecutively treated for classical infection presentation present the same type of immune deficit. The analogy with animal models seems difficult to establish but it appears logical to think that partially missing or inefficient cells involved in immune response to an aggression by *Pasteurella* spp. will favor infection. As in animal models, an increased CD8+ T lymphocytes count should be observed in humans, in reaction to bacterium. However, it is only possible if these cells are sufficient in number and with the possibility to react. Unfortunately, we were not able to challenge CD8+ T cells of patients with their bacterial strain, to observe an activation of these latter.

In conclusion, we are aware that this study does not demonstrate the link between T CD8+ lymphocytes deficit and the occurrence of infection with *Pasteurella* spp., but our observations question the nature of immunity against *Pasteurella* spp. and the likely role of CD8+ T cell lymphocytes. Yet, other studies will be necessary to understand whether only people with a deficit can develop such an infection and to study the exact role of CD8+ T lymphocytes. However, such a study is difficult as many factors are involved such as antibiotic use before cares and bacteriological samples, the presence/absence of the bacteria in animals' mouth and its transmission.

## Data Availability Statement

The raw data supporting the conclusions of this article will be made available by the authors, without undue reservation.

## Ethics Statement

Written informed consent was obtained from the patient for the publication of any potentially identifiable images or data included in this article.

## Author Contributions

ED: proposed the CD8+ T lymphocyte dosage, analyse patient's files, drafted the manuscript, and approved the final version. FF and MA: did the surgery and approved the final version. CM: performed bacterial culture, retrieved patient with previous *Pasteurella* spp. infection, and approved the final version. All authors contributed to the article and approved the submitted version.

## Conflict of Interest

The authors declare that the research was conducted in the absence of any commercial or financial relationships that could be construed as a potential conflict of interest.
